# From west to east; experience with adapting a curriculum in evidence-based medicine

**DOI:** 10.1007/s40037-012-0029-9

**Published:** 2012-10-19

**Authors:** Indah S. Widyahening, Geert J. M. G. van der Heijden, Foong Ming Moy, Yolanda van der Graaf, Sudigdo Sastroasmoro, Awang Bulgiba

**Affiliations:** 1 Department of Community Medicine, Faculty of Medicine, Universitas Indonesia, Jl. Pegangsaan Timur 16, Jakarta Pusat, 10430 Indonesia; 2 Department of Epidemiology, Division Julius Center for Health Sciences and Primary Care, University Medical Center, Utrecht, the Netherlands; 3 Department of Social and Preventive Medicine, Faculty of Medicine, Julius Centre University of Malaya, University of Malaya, Kuala Lumpur, Malaysia; 4 Center for Clinical Epidemiology and Evidence-Based Medicine, Faculty of Medicine, Universitas Indonesia, Cipto Mangunkusumo Hospital, Jakarta, Indonesia

**Keywords:** Curriculum, Medical students, Under-graduate education, Evidence-based medicine

## Abstract

Clinical epidemiology (CE) and evidence-based medicine (EBM) have become an important part of medical school curricula. This report describes the implementation and some preliminary outcomes of an integrated CE and EBM module in the Faculty of Medicine Universitas Indonesia (UI), Jakarta and in the University of Malaya (UM) in Kuala Lumpur. A CE and EBM module, originally developed at the University Medical Center Utrecht (UMCU), was adapted for implementation in Jakarta and Kuala Lumpur. Before the start of the module, UI and UM staff followed a training of teachers (TOT). Student competencies were assessed through pre and post multiple-choice knowledge tests, an oral and written structured evidence summary (evidence-based case report, EBCR) as well as a written exam. All students also filled in a module evaluation questionnaire. The TOT was well received by staff in Jakarta and Kuala Lumpur and after adaptation the CE and EBM modules were integrated in both medical schools. The pre-test results of UI and UM were significantly lower than those of UMCU students (*p* < 0.001). The post test results of UMCU students were comparable (*p* = 0.48) with UI, but significantly different (*p* < 0.001) from UM. Common problems for the modules in both UI and UM were limited access to literature and variability of the tutors’ skills. Adoption and integration of an existing Western CE-EBM teaching module into Asian medical curricula is feasible while learning outcomes obtained are quite similar.

## Background

To be able to provide ‘best practices’, all health care professionals should be able to practice evidence-based medicine (EBM). This requires that medical decisions are based on the best available, current, valid and relevant evidence. In order to do that, medical graduates should ‘be able to gain, assess, apply and integrate new knowledge and have the ability to adapt to changing circumstances throughout their professional life’ [1, 2]. Nowadays many medical schools around the world have incorporated EBM teaching programmes into their curriculum [3]. However, reports on EBM teaching for undergraduate medical students in Asian countries are limited [4–8].

The Sicily statement on teaching evidence-based practice recommends to incorporate knowledge, skills and attitudes of EBM into medical training [1]. However, developing a new curriculum is a challenging task which draws heavily on resources. An alternative is to adopt established EBM and clinical epidemiology (CE) modules and adapt these before implementing them in the existing curriculum.

The Asia link clinical epidemiology and evidence-based medicine (CE-EBM) [9] aims to improve the level of knowledge and skills in CE and EBM of medical students, medical doctors, clinical researchers and other health care professionals in Indonesia and Malaysia. The activities include incorporating CE and EBM teaching in the undergraduate medical curriculum in both the University of Indonesia (UI) and the University of Malaya (UM).

Currently, little is known about the effects of the ‘adapt to adopt’ approach used in curriculum adaptation with regard to EBM and CE teaching. Many papers on EBM and CE teaching report comparisons of different methods of teaching, usually within one medical school. This report describes the process of adapting the existing Utrecht CE and EBM modules and the implementation in an Indonesian and Malaysian curriculum and reports some preliminary outcomes in three medical schools (UI, UM and UMCU). It is also aims to illustrate how some challenges and differences were addressed during the implementation.

## Methods

### The clinical epidemiology and evidence-based medicine (CE-EBM) module

The University Medical Centre Utrecht revised its medical school curriculum in 1999. This involved development of a clinical epidemiology (CE) module and an evidence-based medicine (EBM) module. The Utrecht CE module is a 6-week full-time module targeted to third-year students, which is also the year of their first clinical rotation. The Utrecht EBM module is a 6-week part-time module for sixth (final) year students. During that sixth year, students also have their clerkship. Table 1 presents the content and aim of the Utrecht’s CE and EBM modules.

**Table 1:** Content of the CE and EBM module

Topic	Methods	Module^a^
Introduction to evidence-based medicine	Lecture	EBM
How to make an evidence-based case report	Lecture	EBM
Making an answerable clinical question	Lecture	EBM
Frequency and association measures	Lecture	CE
Diagnosis	Lecture	CE
Prognosis	Lecture	CE
Formulation of the clinical question; search and comparison of study-book knowledge, study of EBCR design	Group work	EBM
Intervention	Lecture	CE
Aetiology	Lecture	CE
Critical appraisals	Lecture	EBM
Internet literature search	Practical lecture	EBM
Computer exercise on prognosis and diagnosis	Practical lecture	CE
Exercise critical appraisal on therapy	Group work	EBM
Establishing the search query, literature search and selection, critical appraisal of the selected papers regarding their relevance and methods, description of methods and (provisional) results	Group work	EBM
Levels of evidence	Lecture	EBM
Computer exercise on aetiology and intervention	Practical lecture	CE
Exercise critical appraisal on prognosis and diagnosis	Group work	EBM
Sorting and structurally summarize the search and appraisal; description of methods and (provisional) results	Group work	EBM
Exercise critical appraisal on aetiology	Group work	EBM
Report of EBCR both orally and in writing according to the requirements for form, structure and content; formulation of a recommendation for patient care and further research	Group work	EBM

^a^Based on the original structure in UMCU where the CE and EBM were given in separate module

Medical training in the three medical schools differs. At UMCU it comprises a 3-year preclinical Bachelor’s and a 3-year clinical Master’s programme; at UI it takes three preclinical years and two clinical years; at UM it takes two preclinical years and three clinical years of training. Therefore, it was not possible to copy and implement the UMCU modules. It was decided to merge and integrate the Utrecht CE and EBM modules. The new module was developed through intensive discussion between the coordinators of the CE and EBM modules of UMCU, UI and UM. It was decided to translate all teaching materials (originally in Dutch) into English. A native speaker assisted this translation and checked the final versions. At UM, English was already the language of teaching but not at UI. Still, it was decided to familiarize the Indonesian students with English as the most-used language in medical literature.

In UMCU both the CE module and EBM modules were coordinated by the clinical epidemiology division of the Julius Center of the Health Sciences and Primary Care, who have approximately 8 years of experience in teaching this module. Clinicians from various departments are also involved as tutors in group work. In UI, the lecturers are clinicians or non-clinicians who have formal training in clinical epidemiology or EBM. Some have more than 5 years’ experience in conducting EBM courses while the tutors are clinicians who have participated in the CE or EBM course. In UM, the lecturers are staff of the Social and Preventive Medicine Department who are experienced in teaching epidemiology and biostatistics. The tutors are also clinicians; however some have never participated in CE or EBM courses.

Before, during and after implementation of the module, personal teaching experiences and evaluation results were shared and discussed, both in an informal and a formal manner. First of all, to familiarize the local staff later involved in teaching the module, a training of teachers (TOT) in UI and UM was led by two experienced lecturers and the module developers from UMCU. This TOT was conducted for 3 days and consisted of lectures and computer practice. In addition, there were regular formal discussion meetings between teachers teams (lecturers, tutors and coordinators) within each university and between the module coordinators of UI (ISW), UMCU (GvdH) and UM (MFM). At the end of each meeting, action points for implementation and optimization of the modules were articulated whenever necessary.

The CE-EBM module in UI was given as a four-week module (condensed) at the end of their fourth year. In UM, it was conducted dispersed within the 3-month period of the social and preventive medicine (SPM) module for third-year medical students. The time allocated for the CE-EBM within the SPM module is a 3-h session twice a week. Moreover, the SPM module is held in a remote satellite campus. Before attending the CE-EBM module, students have passed a research module (UI) or epidemiology and biostatistics module (UM). The comparison of the CE-EBM module structure in the three medical schools is presented in Table 2.

**Table 2:** Comparison of the CE-EBM module structure in UI, UM and UMCU

Educational activities within the module	Time allocation (h)			
	UI	UM	UMCU	
			CE	EBM
Lectures	30	22	20	20
Computer practical	6	8	10	3
Tutorial working group	18	14	5	8
Collaborative and individual learning^a^	45	0	110	55
Plenary presentation	4 × 3	2 × 3	4 × 3	4 × 3
Total hours	111	50	157	98
Module duration	4 week	3 months	6 weeks	5 weeks

^a^Specifically allocated within the module as listed in the schedule

A series of lectures on the diagnostic, prognostic, therapeutic (intervention), and aetiological research were given to re-orientate students on the research design methods relevant to the CE-EBM module. These were further reinforced through computer practice. Lectures introduced students to EBM and the specific skills needed; notably, attention was also given to formulating clinical questions and searching the literature. Critical appraisal skills for relevance and risks of bias and summarizing evidence were taught in small working groups.

The final task of the students was to develop an evidence-based case report (EBCR). An EBCR summarizes the best available evidence and translates it into practice. It follows an explicit and transparent approach to identify such evidence and thereby can help resolve a dilemma in decision-making in real-life patient management. It can be applied at all stages of patient care, notably diagnosis, prognosis and treatment [10, 11].

Students developed their EBCR based on a real clinical patient scenario they had encountered during a previous clinical rotation in tutor-supervised small student groups (*n* = 5). Once a week (in UI and UMCU), the progress in EBCR development was reported in a plenary presentation. These plenary presentation sessions were moderated by experts in clinical epidemiology and EBM, who provided students with feedback. Through these plenary presentations, students were able to learn about different clinical problems of other groups and the appropriate method(s) of dealing them. Due to constraints of the existing curriculum at UM, the plenary presentations were conducted twice during the 3-month module: the first to report the clinical question and the second to report the final EBCR.

### Student assessment

As formative evaluation, students completed a pre- and post-module knowledge test. This test consisted of 32 multiple-choice questions (MCQ) regarding concepts of aetiological, diagnostic, prognostic, therapeutic research and frequency measures. Most of the questions have yes or no answering options, some have five options. In UMCU, this test is used to evaluate the CE module.

EBCRs were assessed using a standard scoring form. The form was based on principles of explicit and transparent reporting of evidence summaries, and included: question, information search, study assessment, data extraction, data synthesis, conclusion, and discussion. The EBCR grades, on a scale of 1–5 (insufficient, doubtful, sufficient, good or excellent), were based on the overall impression of assessors. A student passed the EBCR assignment with a score equal to or above three (sufficient, good and excellent). Ratings for the above separate criteria were also provided as feedback on the aspects that could be improved, with a comment for three categories with the lowest ratings and the highest ratings. The overall scores were multiplied by two to comply with the standard ten-point rating scale used at UI and UM. At UMCU and UI the EBCRs were rated by tutors of working groups, while at UM this was done by the module coordinator.

At the end of the CE module at UMCU and the CE-EBM module at UI and UM a summative evaluation was taken by asking the students to assess the quality of one research article. Students completed a module evaluation questionnaire including questions on the quality of teachers, content and organization of the module.

### Analysis of student assessment data

The knowledge test score was converted into a 1–100 score by computing the percentage of correct answers assuming that all questions have similar weight. The scores between universities were compared using an independent *t* test. ANCOVA was performed to compare the knowledge test score before and after the module, with control for differences in the pre-test results. All analyses were performed using the SPSS 11.0 (SPSS Inc., Chicago, IL).

## Ethical considerations

The CE-EBM module implemented in UI and UM was to become part of the medical curriculum and the effect assessment became part of module evaluation which should be undertaken by all the students. As such, the managerial considerations on the curriculum revision dominated over possible ethical considerations. However, all data were analysed and reported anonymously.

## Results

We evaluated the results of the module implementation in 2010. In UI, the CE-EBM module was conducted from May to July in two rotations with a total of 202 students. In UM, the module was implemented from September until December 2010 for 200 students. The EBM module in UMCU was conducted throughout the year, divided into six groups; a total of 381 students participated.

### Student assessment

Table 3 shows the results of the pre- and post-module knowledge assessment and EBCR scores at UI, UM and UMCU. The mean pre-test results of the UI students were significantly lower than those of the UMCU (*p* < 0.001). After the module, the improvement (change) of knowledge in UI students was significantly higher than for the UMCU students (*p* < 0.001), which resulted in comparable post-test results (*p* = 0.484). On the other hand in the UM, even though the improvement (change) of knowledge of the students was also significantly higher than at UMCU (*p* < 0.001), the post-test results were still significantly lower than for the UMCU (*p* < 0.001). The mean EBCR score of UI students was significantly higher (*p* = 0.001) compared with both UMCU and UM. Examples of some of the clinical problems answered by EBCR are presented in Table 4.

**Table 3:** Comparison of pre- and post-knowledge test results and evidence-based case report (EBCR) score between UI, UM and UMCU

	UMCU	UI	UM
Pre-test scores			
N	389	196	160
Mean (SD)	62.20 (9.21)	54.69 (10.51)	46.23 (8.69)
Minimum	9.38	31.58	28.12
Maximum	87.50	92.11	68.75
Mean difference (95 % CI)		7.52 (5.85–9.18)	15.97 (14.30–17.64)
*p* value		<0.001*	<0.001*
Post-test scores			
N	377	196	200
Mean (SD)	74.75 (9.66)	73.54 (10.13)	61.39 (10.39)
Minimum	31.25	34.21	18.75
Maximum	100	89.48	81.25
Mean difference (95 % CI)		1.32 (0.40–3.04)	13.36 (11.66–15.06)
*p* value		0.484†	<0.001†
Difference in scores			
N	350	196	159
Post-test minus pre-test	12.54 (11.44)	18.85 (12.81)	17.51 (9.49)
Mean difference (95 % CI)		6.32 (4.22–8.41)	4.98 (2.93–7.02)
*p* value		<0.001*	<0.001*
EBCR			
N	71	40	40
Median	8.0	8.4	7.8
Minimum	5	7.5	7
Maximum	10	9.0	8.6
*p* value		0.001‡	0.44‡

* Independent *t*-test

† Ancova test, which include baseline score as covariate

‡ Mann–Whitney test

**Table 4:** Examples of clinical questions answered by evidence-based case reports

Clinical questions	Type of question
University of Indonesia	
Is artemether–lumefantrine (AL) as effective as artesunate–amodiaquine (ASAD; standard treatment of malaria therapy in Indonesia) for treating uncomplicated childhood malaria?	Therapy
Mortality after balloon aortic valvuloplasty in male with severe aortic stenosis	Prognosis
Is the nitrite test accurate as diagnostic tool in pregnant women?	Diagnosis
In hospitalised elderly patients, is depression related to higher mortality?	Prognosis
Could calcium supplementation prevent osteoporotic fracture in post-menopausal women?	Therapy
Diagnostic value of abdominal radiography to diagnose acute appendicitis in children	Diagnosis
The effectiveness of normal saline solution vs Ringer’s lactate solution to overcome dengue shock syndrome in children	Therapy
Is acupuncture effective to decrease pain in patient with chronic back pain?	Therapy
University of Malaya	
Is exercise stress echocardiography superior in diagnosing patients presenting with chest pain compared with exercise stress electrocardiography?	Diagnosis
Can Parkinson’s disease be treated more effectively by using dopamine agonists compared with levodopa in Parkinson patients above 50 years?	Therapy
Do colorectal cancer patients with high levels of tumour markers CEA and CA19-9 have a low 3-year survival rate?	Prognosis
Does the Revised Trauma Score (RTS) make a good prognostic tool in trauma patients?	Prognosis
Do children with past history of sexual abuse have increased risk of psychiatric problems in adulthood?	Prognosis
University Medical Center Utrecht	
The prognostic value of a history of shoulder pain on predicting the duration of pain in patients presenting with a new episode of shoulder pain	Prognosis
The influence of physiotherapy on pain in patients with shoulder impingement	Therapy
Diagnostic ultrasound in a patient suspected of a partial rotator cuff rupture	Diagnosis
Does treatment with heparin in adults diagnosed with cerebral venous thrombosis reduce mortality within 3-months?	Therapy
CT venography: the new reference test for diagnosing cerebral venous thrombosis?	Diagnosis
Glasgow Coma Scale as a prognostic factor in sinus thrombosis	Prognosis
Prognostic value of current smoking for exacerbations in COPD	Prognosis
For patients with COPD in the primary care setting: what is the predictive value of CRP for community-acquired pneumonia?	Prognosis
Diagnostic value of BNP for determining chronic heart failure in patients with COPD	Diagnosis

### Module evaluation

The three medical schools have a different module evaluation format, thus no statistical testing was performed to compare the results. In general, more than 80 % of students in UI and UMCU agreed that the module increased their knowledge and skills as shown in Table 5. Students especially appreciated the searching and critical appraisal skills they learnt through this module which they believed to be very useful for their future.

**Table 5:** Module evaluation by students at the end of the module

	Proportion of students agree (%)		
	UI	UMCU	UM
The objective of the course is achieved	89	N.A.	48.7
The course increased my competence (knowledge and skills)	85	95	N.A.
The course is useful for my professionalism	N.A.	60	56

Only 49 % of UM students agreed that this module had achieved its objectives as compared with 89 % in UI. Several problems were mentioned by students in the module evaluation as well as reported by module coordinators in both UI and UM including limited access to literature (UI), inconsistent internet connection as students were located in a remote satellite campus (UM) and the variability of the tutors’ skills despite the TOT (UI and UM). To help the students retrieve some articles which are not accessible through the current library access at UI, tutors or module coordinators sometimes needed to contact their colleagues in other institutions with better library access (both inside and outside the country) who could provide the articles. The variability in tutors’ skills was handled by providing adequate time for discussion among tutors and resource persons during the module through regular meetings and electronic communication. Besides that, both UI and UM students thought that the searching skills should have been introduced earlier.

## Discussion

This report shows that implementation of an adopted CE-EBM curriculum from a Western country (the Netherlands) in Eastern countries (Indonesia and Malaysia) resulted in a comparable increase in medical student knowledge. The introduction of a CE-EBM module as part of the medical curriculum is needed as a response to global development. Adaptation of an established module was chosen as a more practical approach instead of developing a new one. Yet, it still has its own challenges. A modification process which comprises broad aspects such as learning objectives, resources, institutional mandates and values needs to be carried out to provide more context [12].

A survey about EBM teaching in UK medical schools has identified similar challenges including the need for standard curriculum and teaching materials, the lack of people trained in EBM to facilitate small group sessions, and also the need for an identifiable coordinator who is responsible for the development and integration of EBM into the medical school curriculum [13]. Our experience shows that those challenges could be minimized using this ‘adapt to adopt’ approach.

### Learning goals

EBM is defined as the uptake and use of best available evidence, and its integration with clinical expertise and patients’ values, in circumstances of individualized patient care. The underlying assumption in the definition of EBM is that all aspects on the principles, knowledge and skills needed for this are mastered. The modules used at UMCU, UI and UM are based on the principles of information mastery included in the Sicily statement on teaching evidence-based practice [1]. In the context of self-directed and problem-based learning this implies that we aim for the third level of Miller’s pyramid [14].

### Student assessments

To determine the effect of a curriculum intervention, a prospective pre-test-post-test controlled trial is the strongest study design [12]. The results of knowledge tests during the CE module at the UMCU and the CE-EBM module test at UI and UM were compared. Despite the research module (UI) and epidemiology and biostatistics module (UM) attended prior to the CE-EBM module, the scores of the UI and UM students during pre-test were significantly lower compared with those of UMCU students. It seems that both previous modules at UI and UM focused more on the study design (i.e. cross-sectional, case–control, cohort and experiment) and bio-statistics rather than the clinical epidemiology approach (diagnostic, prognostic, therapeutic and aetiological).

A standard scoring form was used for EBCR assessment, but there was no training or standardization of these assessments. Differences in the EBCR ratings may be explained by different raters and variation among them. While at UM there was only one rater, the Jakarta tutors may—compared with the UMCU raters—have been too unfamiliar to this kind of report. This may in part explain the negatively skewed distribution of EBCR ratings at UI.

Students knew that the MCQ test scores served as a formative evaluation, so these were not included in the module grading. We did not apply correction for guessing for the MCQ test. Since all questions were to be completed and no answers could be left blank, guessing might result in both higher or lower scores [15]. Still, an increase in MCQ score was observed.

The knowledge test was developed based on the predefined module learning goals, and thus determined the content and face validity of the test as educational measurement tool. The test outcomes presented may, however, represent a mix of results based on the quality of the module [16], teachers [17] and students [18]. A formal psychometric evaluation of the reliability has not yet been performed.

Although a formal psychometric evaluation of the student assessment tests may deepen insight into their quality and may perhaps provide a more detailed view on the reported differences between UMCU, UI and UM in learning outcomes, it is not the purpose of our paper to show the quality of the tests used. But given the content and face validity of the tests used we consider these appropriate to illustrate the success of the adoption of the CE-EBM module at UI and UM medical schools.

### Schools compared

Despite a lot of differences between the three medical schools, the post-module results show that similar effects were achieved on students’ knowledge and skills in UI and UMCU. In design and organisation the UI module more closely resembled the original Utrecht teaching modules. The full-time 4-week CE-EBM module in year 4 at UI resulted in comparable score with UMCU, which ran a separate full-time 5-week CE module in year 3 and a 5-week part-time EBM module in year 6.

Due to cultural differences in the approach to teaching and learning and in health care systems, it was expected that the use of Western teaching material and approach in the Asian context and environment may pose particular challenges. This may have played a role in the success of this curriculum revision at UM and UI. Indonesia and Malaysia are still considered to be developing countries. Several obstacles to teaching and practising EBM in the developing world have been identified, including limited resources, limited access to databases and libraries, and lack of role models [8]. Those factors need to be considered during the process of adapting the curriculum from a Western developed country (the Netherlands) to UI and UM. Still, since 2012 the module has proved to be a success at both UI and UM and is still in place.

From the start, the UM module had a more complex environmental and organisational structure. The differences in the module implementation were probably too abundant in UM thus similar results were not being observed. This is also corroborated with the result of the module evaluation which showed that a great number of the UM students felt that this module failed to achieve its objectives. Moreover, students and teachers at UI and UM were quite used to a problem-based learning (PBL) or a self-directed learning approach. For teaching EBM, however, directed learning including lectures followed by a group tutorial may be more effective than PBL [6].

### Study limitations

Systematic differences in the structure, time, approach of the module and a difference in their place in the curricula of the three medical schools could not be controlled for. Some differences in the tools used for student assessments (i.e. module evaluation questionnaire, EBCR ratings) may have had an impact on our findings. Despite these limitations, the measured objective and standardised outcomes in the large number of participating students in a multi-institutional comparison contribute to the robustness of the findings of our study [19].

## Conclusions

Adoption of an existing Western EBM module into Asian medical curriculums is feasible and more practical than developing a new one. Despite differences in the programmes and the students, similar learning goals can be reached in different ways. Our experience could be used by other medical schools in Asian countries to start teaching EBM to their medical students.

## Essentials

- To introduce clinical epidemiology and evidence-based medicine teaching to medical students, adaptation of an established module from another medical school is more practical than developing a new one.
- The adaptation process should consider the broad aspect of the local situation including institutional values, resources, structure and organization of the existing medical school curriculum.
- Several contributing factors for successful implementation of the module are initial capacity building for teachers (training of teachers), continuous team discussions and feedback to the teachers, mentoring from the original module developers, and a dedicated module coordinator who is supported by the management of the medical school.
